# Pattern of microbial translocation in patients living with HIV-1 from Vietnam, Ethiopia and Sweden

**DOI:** 10.7448/IAS.17.1.18841

**Published:** 2014-01-24

**Authors:** Samir Abdurahman, Babilonia Barqasho, Piotr Nowak, Do Duy Cuong, Wondwossen Amogne, Mattias Larsson, Lars Lindquist, Gaetano Marrone, Anders Sönnerborg

**Affiliations:** 1 Division of Clinical Microbiology, Department of Laboratory Medicine, Karolinska Institute, Karolinska University Hospital, Stockholm, Sweden; 2 Department of Medicine, Unit of Infectious Diseases, Karolinska Institutet, Karolinska University Hospital, Sweden; 3 Infectious Diseases Department, Bach Mai Hospital, Hanoi, Vietnam; 4 Department of Medicine, Faculty of Medicine, Addis Ababa University, Ethiopia; 5 Division of Global Health (IHCAR), Department of Public Health Sciences, Karolinska Institutet, Stockholm, Sweden; 6 Oxford University Clinical Research Unit (OUCRU), Hanoi, Vietnam

**Keywords:** microbial translocation, immune activation, LPS, sCD14, treatment-naïve patients living with HIV, HIV in Vietnam, HIV in Ethiopia, HIV in Sweden

## Abstract

**Introduction:**

The role of microbial translocation (MT) in HIV patients living with HIV from low- and middle-income countries (LMICs) is not fully known. The aim of this study is to investigate and compare the patterns of MT in patients from Vietnam, Ethiopia and Sweden.

**Methods:**

Cross-sectional samples were obtained from treatment-naïve patients living with HIV-1 and healthy controls from Vietnam (*n*=83; *n*=46), Ethiopia (*n*=9492; *n*=50) and Sweden (*n*=51; *n*=19). Longitudinal samples were obtained from a subset of the Vietnamese (*n*=24) in whom antiretroviral therapy (ART) and tuberculostatics were given. Plasma lipopolysaccharide (LPS), sCD14 and anti-flagellin IgG were determined by the endpoint chromogenic Limulus Amebocyte Assay and enzyme-linked immunosorbent assay.

**Results:**

All three biomarkers were significantly increased in patients living with HIV-1 from all countries as compared to controls. No differences were found between males and females. Vietnamese and Ethiopian patients had significantly higher levels of anti-flagellin IgG and LPS, as compared to Swedes. ART reduced these levels for the Vietnamese. Vietnamese patients given tuberculostatics at initiation of ART had significantly lower levels of anti-flagellin IgG and higher sCD14. The biomarkers were lower in Vietnamese who did not develop opportunistic infection.

**Conclusions:**

Higher MT is common in patients living with HIV compared to healthy individuals, and in patients from LMICs compared to patients from a high-income country. Treatment with tuberculostatics decreased MT while higher levels of MT are associated with a poorer clinical outcome.

## Introduction

Gut–blood barrier dysfunction is a hallmark of human immunodeficiency virus type 1 (HIV-1) infection. It is associated with translocation of microbial products, thereby contributing to the systemic immune activation seen in patients living with HIV-1 [1, 2]. In diseases such as Crohn’s disease and ulcerative colitis, bacterial flagellin has been pinpointed to be of major pathogenetic importance, and high plasma levels of immunoglobulins (IgG) directed towards this bacterial antigen are present [3–5]. We and others have reported increased plasma levels of microbial translocated products in patients living with HIV-1 who live in high-income countries, which decrease during antiretroviral therapy (ART) [6, 7]. Also, we have suggested that various drug combinations may have different outcomes in terms of improvement of the microbial translocation (MT) markers in patients living with HIV-1 [8]. In contrast, the role of MT in the HIV epidemic in low- and middle-income countries (LMICs) remains controversial and not well studied. It has been suggested that MT is important for HIV disease progression in Africa [9], but this has been disputed [10]. Also, increased MT has been reported in South African subjects before ART, but it remained elevated during efficient therapy [11].

In order to obtain more knowledge about MT in LMICs, we sought to evaluate to which extent Vietnamese and Ethiopian patients living with HIV-1 demonstrate higher levels of biomarkers for MT and if efficient ART influenced these levels in the Vietnamese patients.

## Methods

### Study populations

The study populations were obtained from three countries: Vietnam, Ethiopia and Sweden (Table 1). The Vietnamese patients were part of a prospective, longitudinal study, and detailed clinical information as well as follow-up samples were available. The blood samples were drawn before starting ART and at 24 months after initiation of ART. For the Ethiopian patients, only a cross-sectional analysis was performed due to the lack of follow-up data. Swedish patients were analyzed in a cross-sectional fashion for comparison with the Vietnamese and Ethiopian patients. Both the Ethiopians and the Swedes were untreated.

**Table 1:** Characteristics of Vietnamese, Ethiopian and Swedish patients living with HIV-1

Characteristics	Vietnamese patients	Ethiopian patients	Swedish patients	*p*^a^
*N*	83	92	51	–
Age (years) (median, IQR)	35 (32–38)	35 (29–41)	38 (30–46)	0.044
Sex (*n*) (%)	–	–	–	<0.001
Female	19 (23)	65 (70)	26 (48)	–
Male	64 (77)	27 (30)	25 (52)	–
Baseline CD4+ T-cells (cells/µl) (median, IQR)	64 (24–169)	100 (56–144)	212 (138–280)	<0.001
Baseline HIV viral load [log_10_ copies/ml (median, IQR)]	4.8 (4.3–5.3)	5.4 (5.0–5.8)	5.4 (5.0–5.8)	<0.001
BMI (kg/m^2^)	19 (18–20)	20 (18–23)	ND	0.005
Mode of HIV infection (*n*) (%)	–	–	–	<0.001
Heterosexual	31 (37)	92 (100)	34 (67)	–
Homosexual	–	–	10 (19)	–
Injecting drug use	38 (46)	–	4 (8)	–
Others or unknown	14 (17)	–	3 (6)	–

a Kruskal–Wallis, Wilcoxon rank sum or chi-squared test as appropriate. IQR, interquartile range; ND, not done; BMI, body mass index.

The Vietnamese patients (*n*=83) (Table 1) were randomly selected from a larger number of subjects (*n*=640) who were part of a randomized controlled trial in northeastern Vietnam between 2008 and 2011, which assessed the effect of peer support on treatment failure (registration number: NCT01433601) [12]. The selected 83 patients had similar demographic and laboratory characteristics as the whole cohort (data not shown). Twenty-four subjects had ongoing tuberculosis (TB) diagnosed two months before inclusion in the study and had been given tuberculostatics for two months (rifampicin, isoniazid, ethambutol and pyrazinamide), with an efficient therapeutic response to the TB therapy, before starting ART [stavudine (d4T) or zidovudine (ZDV) plus lamivudine (3TC) plus nevirapine (NVP) or efavirenz (EFV)]. In six of these subjects, treatment continued for an additional ten months, while remaining 18 subjects received only six months of treatment (isoniazid and ethambutol). Clinical information for all subjects was obtained from their physician during follow-up. All patients had undetectable HIV load at 24-month follow-up. Plasma samples were drawn before ART and in 38 cases at 24-month follow-up, and they were thereafter stored at −80°C. Samples were also obtained from 46 healthy Vietnamese HIV-negative controls who originated from the same socioeconomic grouping and geographical area.

Plasma samples were also obtained from 92 treatment-naïve Ethiopian patients living with HIV-1 (Table 1) from three different health care centres in Addis Ababa. The patients had been assessed clinically by an experienced physician. No signs of TB had been found and no TB treatment had been given. Fifty healthy HIV-negative Ethiopian controls who were matched with regard to socioeconomics, age and sex, all living in Addis Ababa in the same geographical region, were included as controls. No follow-up data with clinical information or follow-up samples were available. From Sweden, 51 treatment-naïve patients living with HIV-1 followed at Karolinska University Hospital were included for comparative purposes (Table 1) along with 19 healthy HIV-negative Caucasian Swedish individuals, all living in the capital city of Stockholm with a good socioeconomic situation. The samples had been stored at −80°C until use. None of patients living in Sweden had an ongoing TB infection as determined by chest X-ray and standard clinical investigations.

The study was conducted in accordance with the Declaration of Helsinki and was approved by the Regional Ethics Committee in Stockholm, Sweden; the Institutional Review Board of Addis Ababa University, Ethiopia; and the Hanoi Medical University Review Board in Bio-medical research, Vietnam.

### Anti-flagellin enzyme-linked immunosorbent assay (ELISA)

An in-house ELISA was developed for the detection of plasma anti-flagellin IgG antibodies. High-binding 96-well microwell plates (MWPs) (Costar) were coated with purified flagellin monomers from *S. typhimurium* and incubated overnight at 4°C. The MWPs were washed and blocked with 1% bovine serum albumin in phosphate-buffered saline (PBS) at room temperature for 1 h. Plasma samples (diluted 1:1000 in PBS) were added to the plates and incubated at room temperature for 2 h. The MWPs were washed and horseradish peroxidase-conjugated anti-human antibody (1:20,000) was added before being incubated further at room temperature for 1 h. Finally, the MWPs were washed and the plates were read in a Labsystems multiscan MCC 340 spectrophotometer.

### Detection of lipopolysaccharide (LPS) and sCD14

Plasma LPS levels were determined using the endpoint chromogenic Limulus Amebocyte Assay (Lonza, Walkersville, MD, USA), essentially as described in the manufacturer’s protocol except that the samples were diluted 1:5 in an endotoxin-free 10 mM MgCl_2_ solution and inactivated for 12 min in 70°C using an endotoxin-free glass tubes. A commercial ELISA kit (R&D Systems, Inc., Minneapolis, MN, USA) was used to measure plasma concentrations of soluble CD14 according to the manufacturer′s recommendations.

### Statistical analysis

Due to the non-normal distribution of the outcomes of interest (anti-flagellin IgG levels, LPS and sCD14), the non-parametric Mann–Whitney *U* test was used to compare median differences between patients and controls at baseline. Intragroup changes from baseline to the end of the study were analyzed by a Wilcoxon test for paired samples. Comparisons of more than two groups were performed using the Kruskal–Wallis non-parametric test. All tests had a 95% confidence level. Correlations between the outcomes and the CD4+ T-cell count, viral load, age, sex, route of infection and body mass index (BMI) were calculated using Spearman’s rank correlation coefficient.

Multivariable linear regression models with bootstrap confidence intervals in order to take into account the non-linearity of the outcomes (tested by the Shapiro–Wilk test for normality) were used to compare baseline differences in anti-flagellin IgG, sCD14 and LPS levels between Vietnamese, Ethiopian and Swedish patients living with HIV-1. Factors considered in the regression model were also age, sex and baseline CD4+ T-cell count. GraphPad Prism software, version 5.04 (GraphPad Software Inc., San Diego, CA, USA) and Stata (Stata Corporation, College Station, TX, USA) were used for these analyses. *P*-values <0.05 were considered significant.

## Results

### Anti-flagellin IgG levels in Vietnamese patients

**Figure 1:**
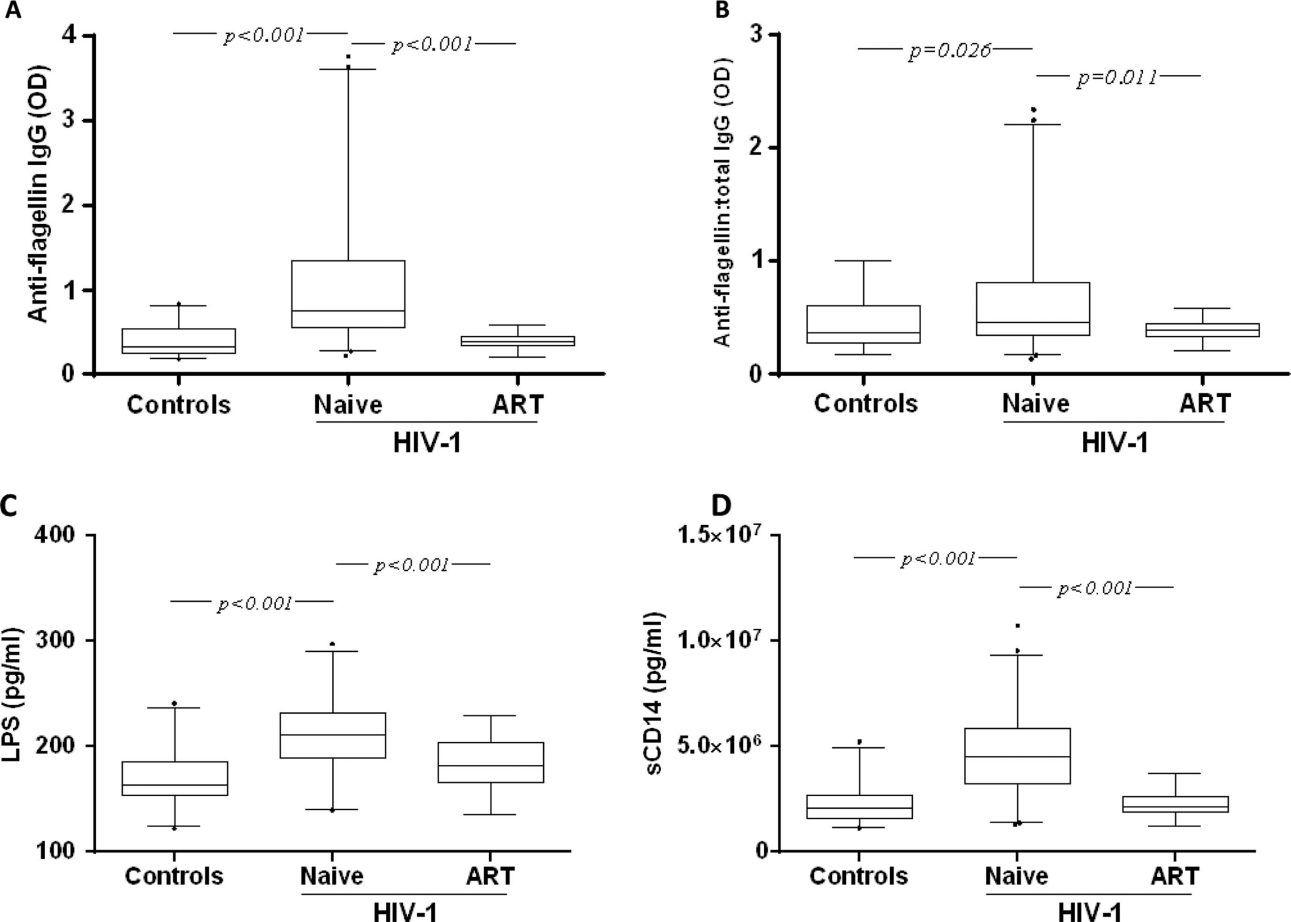
Detection of plasma anti-flagellin IgG, lipopolysaccharide (LPS) and soluble CD14 (sCD14) in Vietnamese patients living with HIV-1 and in HIV-negative controls. Box plot representing the plasma levels of anti-flagellin IgG (A), the ratio of flagellin-specific IgG to total IgG (B), LPS (C) and sCD14 (D) in healthy controls (Controls), naïve individuals living with HIV-1 (Naïve) and individuals living with HIV-1 after 24 months of antiretroviral therapy (ART). Box plots display the median OD value and 25th and 75th interquartile ranges. Whiskers represent the 5th to 95th percentiles, and dots the outliers. The non-parametric Mann-Whitney test was used to compare the differences between patients and controls at baseline. The intragroup change from baseline to the end of the follow-up study was analyzed by a Wilcoxon test.

All Vietnamese subjects (*n*=83) had detectable plasma anti-flagellin IgG. At baseline, the levels were higher in these ART-naïve patients living with HIV-1 as compared with the HIV-negative controls [median optical density (OD): 0.76 (IQR 0.55–1.34) versus 0.32 (IQR 0.25–0.53); *p*<0.001] (Figure 1A). In the patients (*n*=38) in whom follow-up samples were available, the anti-flagellin IgG were reduced by two years of ART to similar levels [median OD: 0.40 (IQR 0.33–0.45); *p*<0.001] as in the controls.

Similar statistically significant differences in the total IgG levels were observed (data not shown). When the flagellin-specific IgG were adjusted to the total IgG, the differences between the patients living with HIV-1 at baseline and the controls persisted [median OD: 0.46 (IQR 0.34–0.82) versus 0.37 (IQR 0.28–0.60); *p*=0.01] (Figure 1B). There was no significant correlation between anti-flagellin IgG with CD4+ T-cell count, viral load, sex, route of infection or BMI. A weak correlation was found with age (*r*=0.28, *p*=0.01).

### LPS and sCD14 levels in Vietnamese patients

At baseline, the plasma LPS and sCD14 levels were higher in the Vietnamese patients living with HIV-1 as compared to the healthy Vietnamese controls [LPS, median pg/ml: 211 (IQR 190–232) versus 163 (IQR 153–185); sCD14, median pg/ml: 4,535,000 (IQR 3,237,000–5,898,000) versus 2,093,000 (IQR 1,577,000–2,724,000); *p*<0.0001 for both] (Figure 1C and 1D). The levels of LPS and sCD14 were reduced following two years of ART [LPS, median pg/ml: 182 (IQR 166–203); sCD14, median pg/ml: 2,116,000 (IQR 1,885,000–2,632,000); *p*<0.0001 for both], although the LPS level was not normalized and remained slightly higher than in the controls [median pg/ml: 163 (IQR 153–185); *p*=0.004] (Figure 1C and 1D). No correlation was found between the levels of LPS and sCD14 and CD4+ T-cell counts, viral load, age, sex, route of infection or BMI, respectively.

### Anti-flagellin IgG, LPS and sCD14 in TB-treated Vietnamese patients

Twenty-four of the Vietnamese participants had received TB treatment for two months prior to starting ART (Table 2).

**Table 2:** Characteristics of Vietnamese patients living with HIV-1, with and without tuberculosis (TB) treatment, at baseline

Characteristics	Patients with TB	Patients without TB
*N*	24	59
Age (years) (median, IQR)	34 (31–39)	35 (33–38)
Sex (*n*) (%)		
Female	3 (13)	13 (22)
Male	21 (87)	46 (78)
Baseline CD4+ T-cells (cells/µl) (median, IQR)	49 (23–103)	79 (23–174)
Baseline HIV viral load [log_10_ copies/ml (median, IQR)]	4.6 (4.4–5.3)	4.7 (4.2–5.2)
BMI (kg/m^2^)	19 (17–20)	19 (18–20)
Hemoglobin (g/dL)	131 (118–143)	127 (110–140)
Mode of HIV infection (*n*) (%)		
Heterosexual	6 (25)	20 (38)
Injecting drug use	15 (63)	21 (41)
Others or unknown	3 (12)	11 (21)

TB, tuberculosis; IQR, interquartile range; BMI, body mass index.

**Figure 2:**
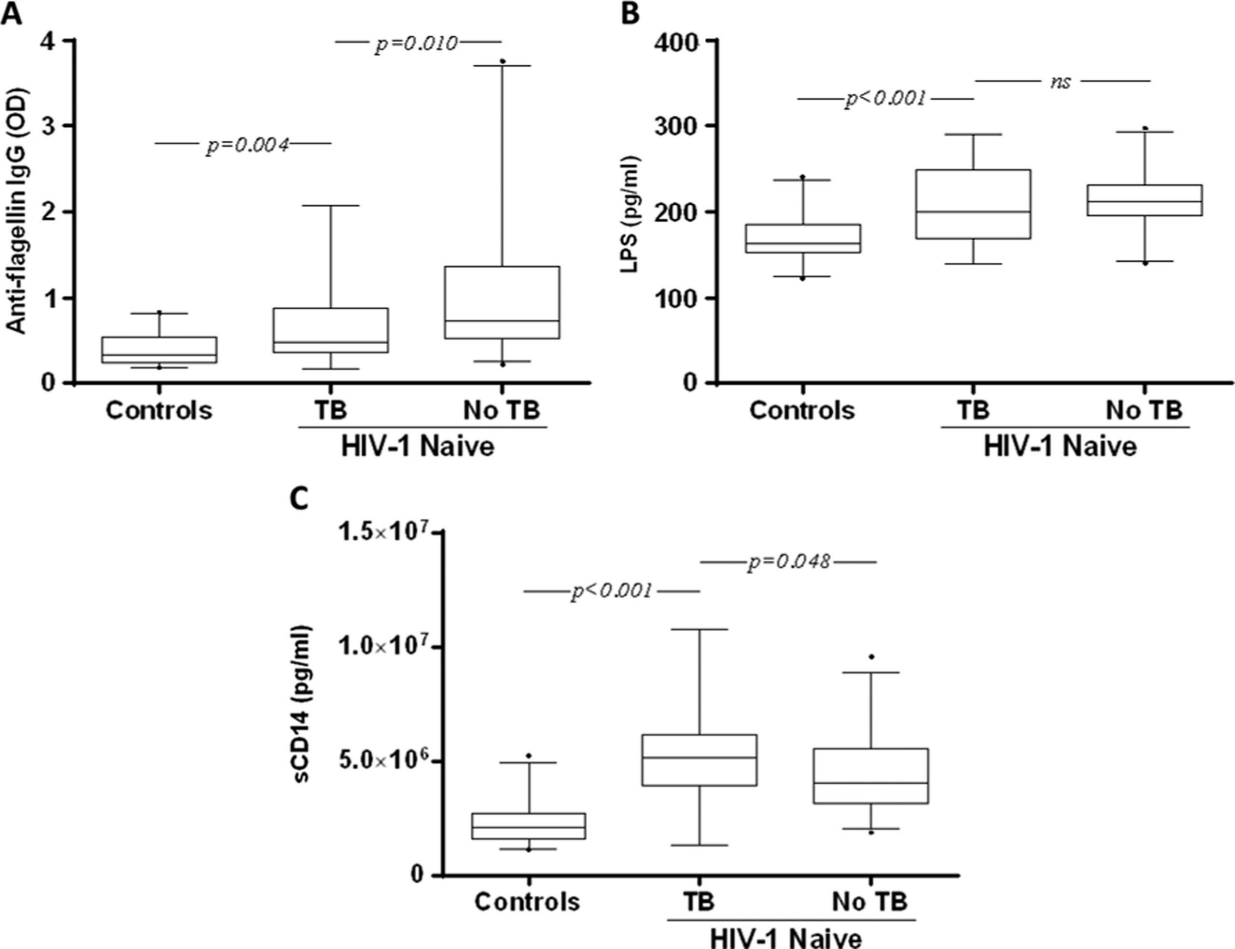
Baseline characteristics of patients living with HIV-1 with and without ongoing tuberculosis (TB) treatment. OD values representing the plasma levels of anti-flagellin IgG (A), LPS (B) and sCD14 (C) in healthy controls (Controls), and naïve individuals living with HIV-1 with (TB) and without (No TB) treatment for TB. Box plots display the median OD value and 25th and 75th interquartile ranges. Whiskers represent the 5th to 95th percentiles, and dots the outliers. Pairwise comparisons refer to a Mann-Whitney test.

The level of anti-flagellin IgG was significantly lower in these subjects as compared to those (*n*=59) without TB treatment [median OD: 0.48 (IQR 0.37–0.88) versus 0.74 (IQR 0.53–1.36); *p*<0.01] (Figure 2A). The level of anti-flagellin IgG in the TB-treated patients was still significantly higher than in the controls [median OD: 0.32 (IQR 0.25–0.53); *p*=0.004].

The level of LPS was lower in the TB-treated patients as compared to those without [median pg/ml: 201 (IQR 169–250) versus 213 (IQR 196–231), respectively], although it was not statistically significant (Figure 2B). Also, the level of LPS in patients given TB treatment was significantly higher than in the controls [median pg/ml: 164 (IQR 153–185); *p*<0.001].

In contrast, the level of sCD14 was significantly higher in TB-treated patients as compared to those without [median pg/ml: 517,100 (IQR 393,200–615,400) vs. 402,400 (IQR 315,000–556,500); *p*=0.048] as well as compared to the controls [209,300 pg/ml (IQR 157,700–272,400); *p*<0.001] (Figure 2C). There were no statistically significant differences between men and women in terms of anti-flagellin (*p*=0.5613), LPS (*p*=0.5805) or sCD14 (*p*=0.6798) levels.

### Anti-flagellin IgG, LPS and sCD14 in Vietnamese patients developing opportunistic infections

The baseline levels of anti-flagellin IgG, LPS and sCD14 were compared between the Vietnamese patients who developed one or more opportunistic infections (OIs) and survived (*n*=27), those who developed a new OI and died (*n*=11) and those who did not develop OIs during follow-up (*n*=45) (data not shown). Patients who had TB treatment at baseline were categorized in the non-OI group (*n*=10), deaths (*n*=9) or a new OI (*n*=14).

**Figure 3:**
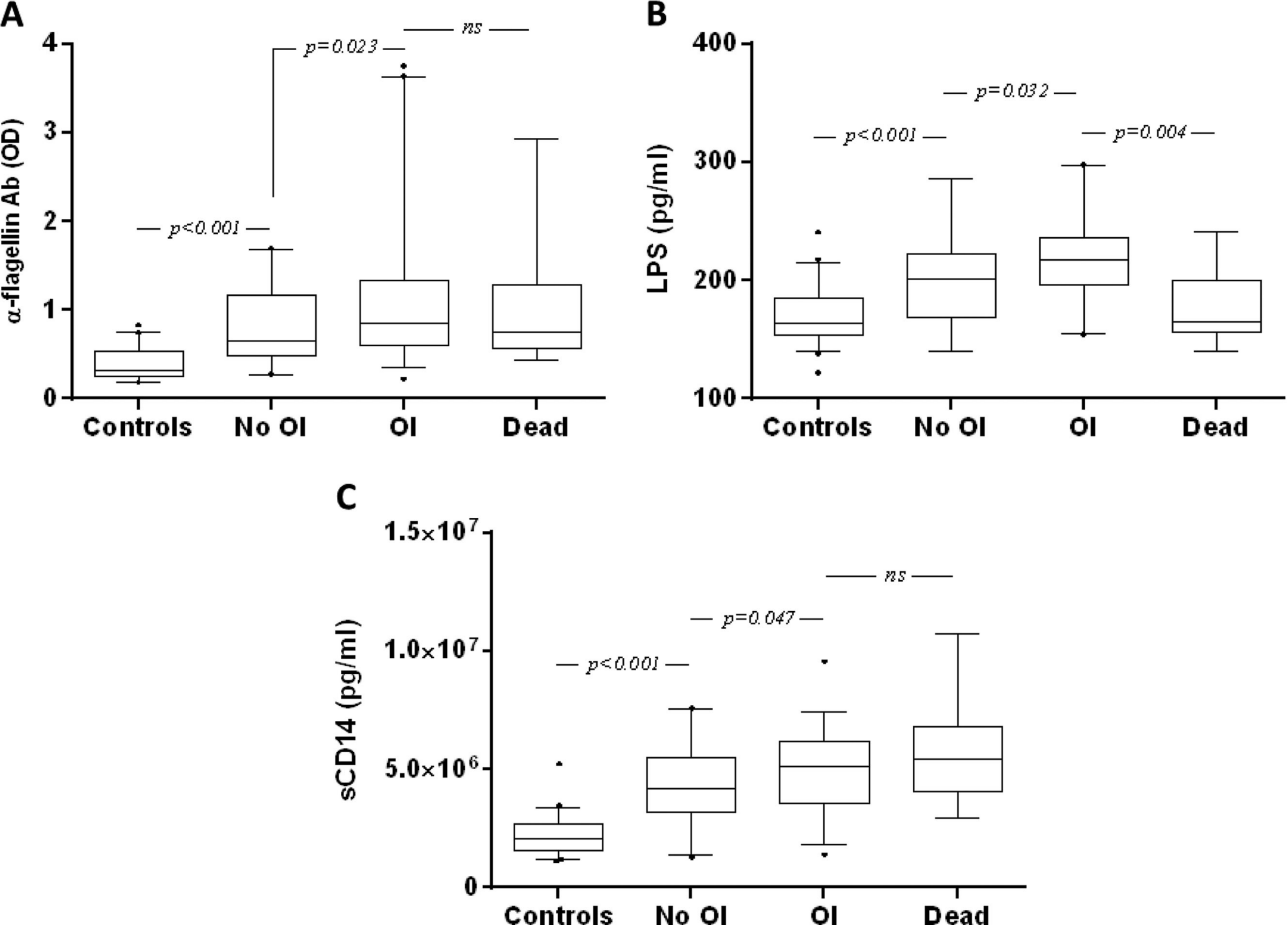
Baseline markers in Vietnamese patients living with HIV-1 with and without new opportunistic infection (OI) development during antiretroviral treatment (ART). Box plot of OD values representing the plasma levels of anti-flagellin IgG (A), LPS (B) and sCD14 (C) concentrations in naïve Vietnamese patients living with HIV-1 who died (Dead), and those with (OI) or without (No OI) the development of OI. Box plots display the median value and 25th and 75th interquartile ranges. Whiskers represent the 5th to 95th percentiles, and dots the outliers. Pairwise comparisons refer to a Mann–Whitney *U* test.

The levels of all three markers were significantly higher in the patients who developed OI and survived, as well as in those who did not develop OI, as compared to the controls (Figure 3).

Also, the levels of anti-flagellin IgG, LPS and sCD14 were significantly higher in patients who developed a new OI and survived as compared to those who did not develop an OI [anti-flagellin IgG, median OD: 0.85 (IQR 0.61–1.34) vs. 0.65 (IQR 0.48–1.16); *p*=0.023; LPS, median pg/ml: 218 (IQR 196–236) vs. 206 (IQR 169–230); *p*=0.032; sCD14, median pg/ml: 5,116,000 (IQR 3,595,000–6,198,000) vs. 4,188,000 (IQR 3,172,000–5,500,000); *p*=0.047] (Figure 3C). We found no statistically significant differences in terms of all three markers between TB/HIV co-infected patients as compared to patients with HIV alone, when charted separately (data not shown). However, patients with TB (and anti-bacterial therapy) had higher risk of developing OI as compared to patients without TB.

In the patients who died, the level of anti-flagellin IgG was not significantly different from that of patients with or without OI, and the levels of LPS were in fact significantly lower as compared to those of patients with (*p*=0.004) or without (*p*=0.049) OI (Figure 3A and 3B). In contrast, the sCD14 levels in patients who died were significantly higher as compared to those of the patients without OI [median pg/ml: 5,459,000 (IQR 4,038,000–6,834,000) versus 4,188,000 (IQR 3,172,000–5,500,000); *p*=0.020]. However, there was no significant difference as compared to patients with OI [median pg/ml 4,188,000 (IQR 3,172,000–5,500,000); *p*=0.310]. We found no statistically significant differences of anti-flagellin (*p*=0.1322), LPS (*p*=0.4350) or sCD14 (*p*=0.3636) levels between men and women with OI.

### HIV load and CD4**+** T-cells in Vietnamese patients

Plasma viral load was significantly higher in the 11 patients who died as compared to the patients with OI who survived and those without OI, respectively [median copies/ml: 230,000 (IQR 82,108–290,000) vs. 82,108 (IQR 29,950–221,848) vs. 37,022 (IQR 15,868–122,570); *p*=0.026, *p*=0.006, respectively]. The level of CD4+ T-cells was lower in patients who died as compared to patients without OI [median cells/ml: 29 (IQR 12–77) vs. 140 (IQR 56–199); *p*<0.001], but not different from that of patients with OI (data not shown).

### Comparisons between Vietnamese, Ethiopian and Swedish patients living with HIV-1

**Figure 4:**
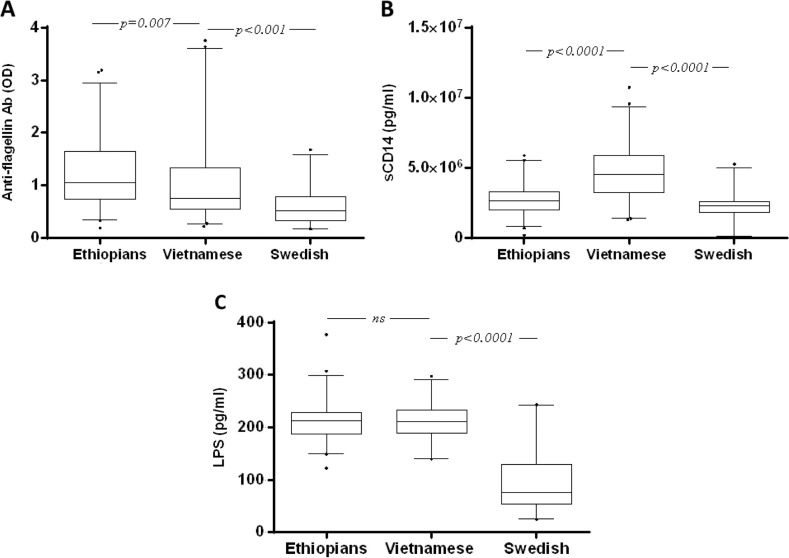
Baseline anti-flagellin antibody levels in treatment-naïve Vietnamese, Ethiopian and Swedish patients living with HIV-1. Box plot of OD values representing the levels of anti-flagellin antibodies (A), plasma sCD14 (B) and LPS (C) concentrations in treatment-naïve Vietnamese, Ethiopian and Swedish patients living with HIV-1. Box plots display the median value and 25th and 75th interquartile ranges. Whiskers represent the 5th to 95th percentiles, and dots the outliers. Pairwise comparisons refer to a Mann-Whitney *U* test.

We compared the level of anti-flagellin IgG, LPS and sCD14 between Vietnamese, Ethiopian and Swedish treatment-naïve patients living with HIV-1 (Figure 4 and Table 3).

**Table 3:** Regression models with bootstrap confidence interval to compare baseline differences in anti-flagellin IgG, sCD14 and LPS levels between Vietnamese, Ethiopian and Swedish patients living with HIV-1 who had no clinical signs of tuberculosis

Outcome	Coefficient (95% CI)	*p*
Anti-flagellin IgG		
Sweden	Ref.	
Ethiopia	0.56 (0.39–0.74)	<0.001
Vietnam	0.39 (0.16–0.61)	<0.01
sCD14		
Sweden	Ref.	
Ethiopia	511,685 (33,864–989,505)	<0.05
Vietnam	2,342,364 (1,675,132–3,009,597)	<0.001
LPS		
Sweden	Ref.	
Ethiopia	139 (119–158)	<0.001
Vietnam	147 (126–168)	<0.001

Results adjusted by CD4 at baseline, age and sex.

In all groups, the levels of anti-flagellin IgG, LPS and sCD14 were significantly higher than in matched HIV-negative controls (data not shown; *p*<0.001 for all). When baseline anti-flagellin IgG levels were compared between the groups using a bivariate analysis, the level in Swedish patients was lower than in both Vietnamese and Ethiopian patients living with HIV (*p*<0.0001 for both). Vietnamese patients also had lower levels of anti-flagellin IgG as compared to Ethiopian patients (*p*=0.007) (Figure 4A). In contrast, sCD14 levels in Vietnamese patients were higher than in both Swedish and Ethiopian patients living with HIV (*p*<0.0001 for both). The sCD14 levels were higher in Ethiopians as compared to Swedish patients (*p*<0.0001). In terms of LPS levels, there was no statistically significant difference between Vietnamese and Ethiopian patients; Swedish patients living with HIV-1 had significantly lower levels of LPS as compared to Vietnamese and Ethiopian patients (*p*<0.0001 for both).

In the multivariable analysis, the age, sex and CD4+ T-cell adjusted differences in all three markers between the groups still persisted, except for the sCD14 differences between Swedish and Ethiopians. The estimated anti-flagellin IgG levels were higher in Vietnamese (*p*<0.0001) and Ethiopian (*p*<0.0001) patients as compared to the Swedes. The Swedish patients were chosen as a reference category, because the Ethiopian patients had a significantly higher expected value of anti-flagellin antibodies (Coeff.=0.54, 95% CI: 0.37–0.72), as did the Vietnamese patients (Coeff.=0.43, 95% CI: 0.20–0.67). The estimated sCD14 levels were significantly higher in Vietnamese patients (*p*<0.0001, Coeff.=2,344,373, 95% CI: 1,778,541–2,910,204) as compared to the Swedes. In contrast, the sCD14 difference between the Swedish and Ethiopian groups was lost and was not statistically significant upon multivariate analysis. In terms of the LPS, the levels were still significantly higher in both Ethiopian (*p*<0.001) and Vietnamese (*p*<0.001) patients as compared to the Swedes: choosing Swedish patients as a reference category, the Ethiopian patients had a significant higher expected value of LPS (Coeff.= 140, 95% CI: 122–156), as did the Vietnamese patients (Coeff.=146, 95% CI: 127–164).

Since it is known that TB may induce increased levels of sCD14 in patients living with HIV-1, we repeated the multivariable analysis after excluding the 24 Vietnamese patients who had ongoing TB (Table 3). Similar results were obtained, although the sCD14 difference also became significant between the Swedish and Ethiopian groups (*p*<0.05) (Table 3).

## Discussion

The present study of patients living with HIV-1 from Vietnam and Ethiopia gives support to the view that MT is an important feature of the HIV-1 disease in LMICs [9]. Thus, the levels of anti-flagellin IgG, LPS and sCD14 were increased in the patients living with HIV-1 from both Vietnam and Ethiopia as compared to HIV-negative controls from the same geographical regions and socioeconomic grouping. Also, patients living with HIV-1 from Vietnam and Ethiopia had higher levels of anti-flagellin IgG and LPS as compared to patients living in Sweden. It can be argued that significant differences existed in, for example, CD4+ T-cells between the patients from the different countries, but the differences also persisted after multivariate analysis (adjusted for age, sex and CD4+ T-cells). It is thus possible that these differences are related to other factors such as different compositions of the gut microbiota and the food intake, poorer hygiene and sanitation and/or frequent exposure to other gastrointestinal infections. Also, another factor that may play an important role for these differences is the viral clade. It is not unreasonable to assume that clade diversity may play an important role in HIV immune activation as a result of MT. Since the viral heterogeneity in our cohorts was limited, the present study did not allow us to evaluate the impact of subtypes on MT. However, it is worth exploring further, and we aim to investigate this aspect in the future.

Higher levels of MT are likely to result in differences in immune activation and thereby to have a profound effect on the host immune profiles afflicting patients living with HIV-1 in LMICs [10, 13, 14]. In contrast to the LPS and anti-flagellin levels, the sCD14 level was highest in the Vietnamese patients, which could be related to the fact that 30% of them were co-infected with TB, which was not the case for the Ethiopian and Swedish patients. Consistent with these observations, Toossi *et al*. found that the level of sCD14 in Ugandan patients living with both HIV and TB was higher than in subjects living with HIV only [15].

It is well established that effective ART reduces MT and immune activation in patients living with HIV-1 who live in high-income countries [1, 16, 17]. The knowledge concerning patients from LMICs is limited to one South African study reporting that increased MT was found before ART and that it decreased but remained elevated during efficient therapy [11]. In our study, the Vietnamese patients, in whom we had follow-up samples, had undetectable virus after two years of ART [12]. A concomitant decrease in the MT markers was seen, suggesting that ART improves the gut–blood barrier damage also found in patients from these surroundings.

A subgroup of the Vietnamese patients (*n*=24) had been treated for two months with tuberculostatics with efficient response. Indeed, we found that these patients had lower anti-flagellin IgG and a tendency to lower LPS. It has been shown that antibiotics decrease the MT in monkeys infected with simian immunodeficiency virus [18], and we have reported that a similar effect is seen in humans living with HIV-1 [8]. It is thus possible that the tuberculostatics had induced changes in the gut microbiota, resulting in a decreased MT. We therefore recommend that any ongoing antibiotic treatment should be considered when MT is studied. In contrast, the sCD14 level was higher than in the patients without tuberculostatics. It is not unlikely that this discrepancy is related to an increased immune activation seen due to remaining disease activity of the TB, since the TB treatment had been given for only two months. Also, we cannot exclude that the increased immune activation was related to other ongoing subclinical OIs. Thus, sCD14 is a biomarker not only for MT but also for other immune activation processes.

We also analyzed the association between the biomarkers and the clinical outcome. As expected, a higher viral load and lower CD4+ T-cells correlated to an increased mortality. Also, the results were consistent with earlier reports noting that higher levels of sCD14 are associated with a poorer clinical outcome [19, 20]. Consistently, patients who did not develop a new OI or died had significantly lower levels of LPS, anti-flagellin IgG as well as sCD14. This finding is in line with the possibility that a more pronounced MT may lead to increased immune activation with an associated poorer immunological response to other infections, including OIs.

In conclusion, we showed that treatment-naïve Vietnamese and Ethiopian patients living with HIV have elevated biomarkers of MT as compared to HIV-negative controls from the same geographical regions and also compared to patients living in Sweden. Also, in the Vietnamese patients the MT was reduced following 2 years of ART. Further investigations of the role of MT in patients living with HIV from LMICs can contribute to an increased understanding of the HIV disease in such countries.

## Conclusions

The role of MT for HIV disease progression in patients living in resource-limited settings is controversial. This study adds more emerging evidence on the prevalence and role of MT in patients living with HIV-1 in low- (Ethiopia) and middle-income (Vietnam) countries being higher than in patients in a high-income country (Sweden).
